# Individual cognitive changes in subjective cognitive decline, mild cognitive impairment and Alzheimer’s disease using the reliable change index methodology

**DOI:** 10.1007/s00508-020-01755-1

**Published:** 2020-10-23

**Authors:** Anna Garcia Rosas, Elisabeth Stögmann, Johann Lehrner

**Affiliations:** Department of Neurology, Medical University of Vienna, Währinger Gürtel 18–20, 1090 Vienna, Austria

**Keywords:** Reliable change index, Neuropsychological testing, Neuropsychological Testbattery Vienna (NTBV), Mild cognitive impairment, Alzheimer’s disease

## Abstract

**Objective:**

The development of Alzheimer’s disease (AD) can be assessed using the neuropsychological test battery Vienna (NTBV). The objective of this study was to investigate whether the NTBV test scores of a diagnostic group have changed significantly over time and whether this change is due to disease progression.

**Methods:**

In this study 358 patients referred to a memory outpatient clinic because of cognitive deterioration were analyzed. The same patients were surveyed in a follow-up assessment after a mean interval of 25.96 months to examine cognitive performance and disease progression. Patients were divided into the subgroups healthy controls (HC), subjective cognitive decline (SCD), mild cognitive impairment (MCI) and AD on the basis of the test results. Reliable change index methodology was used to assess improvement or deterioration in test scores in diagnostic groups compared to HC.

**Results:**

Deterioration in the SCD group ranged from 0% to 18.8%. The MCI group showed declines between 1.6% and 29.1%. Patients who developed AD deteriorated between 0% and 54.2%. Improvements ranged from 0% to 73.4% in the SCD group and from 0% to 25.1% for the MCI group. The improvement in the AD group ranged between 0% and 44.0%.

**Conclusion:**

The results reflect the cognitive deterioration of patients during the disease progression. Nevertheless, improvements in diagnostic groups could be detected. The significantly positive changes might be due to practice effects, also a lack of motivation or attention in the first test could have yielded “improvement” in the retest.

## Key points

- Cognitive changes due to Alzheimer’s disease can be assessed by the neuropsychological test battery Vienna.
- Patients were classified into the groups healthy controls, subjective cognitive decline, mild cognitive impairment and Alzheimer’s disease based on diagnosis criteria.
- According to reliable change index methodology, diagnostic groups showed individual deteriorations as well as improvements in the test scores.
- The deteriorations demonstrate the cognitive impairments in the disease process, while the reason for improvement in test performance was mainly practice effects.

## Introduction

The neurodegenerative disorder Alzheimer’s disease (AD) is often diagnosed in older people [1]. It is characterized by acquired disorders of cognitive performance and emotional control of conscious patients that interfere with everyday activities [2]. Previous studies revealed a typical process of three phases in the development of AD, which starts with the subjective cognitive decline (SCD), continues into mild cognitive impairment (MCI) and ends in AD [3, 4]. The development of AD can be seen in changes in cognitive performances, as measured by test scores on specific neuropsychological tests. The most salient changes in the disease process take place in the episodic memory. In particular, deficits of the verbal and visuospatial memory are both concise signs that have been reported as being related to the development of AD [5–8]. Beside the mentioned cognitive performances, other cognitive parameters, such as naming ability, abstraction, verbal fluency and executive functioning are known to worsen with the progress of the disease [9–11].

Cognitive changes can be assessed by neuropsychological tests. To capture individual changes in test results, the reliable change index (RCI) method, which is determined by the test-retest reliability (rtt), was developed. It analyzes the influence of disease progression over time and calculates the ability of each participant across both examinations. taking into consideration changes not linked to cognitive deterioration. These can be caused by practice effects or measurement errors [12]. If a neuropsychological test is presented to a patient more than once, improvement can be attributed to practice effects: however, age, disease development or therapeutic interventions can also have an influence on test results. This should be considered when interpreting the results [13–15].

The aim of the study was to determine if a diagnostic group had significant changes in test scores over time, which could not be accounted for by systematic or measurement error. Additionally, the RCI methodology was used for comparison between healthy controls (HC) and patients diagnosed with SCD, MCI and AD.

## Methods

### Participants

The current study was based on a dataset that is part of a research project called the Vienna Conversion to Dementia study. The ethics committee of the Medical University of Vienna approved the study. Data from 358 participants, which were collected from 2008 to May 2018, were examined. After an interval of 12–48 months a follow-up examination was done. Before the testing the participants received information about the questionnaires and an elaborated explanation of this study. The surveys were distributed based on the possible cognitive impairment of the participants and their referral to a doctor. All patients underwent standard laboratory blood testing, clinical examination and neuropsychological testing. The patients were subdivided into four groups: HC, SCD, MCI and AD. The diagnosis of SCD was made according to the criteria of Jessen et al. [16], while the diagnosis of MCI was determined by Petersen’s criteria [17]. The DSM‑4 criteria [18] and NINCDS-ADRDA guidelines [19] were used to diagnose AD. The HC group included patients without an active neurological or psychiatric disease or psychotropic medications and no self-experienced persistent decline in cognitive capacity. Participants with a history of cerebral vascular pathology, a diagnosis of AD at the first session and an age under 51 years were excluded from this study. Additionally, participants with head injury and a current psychiatric diagnosis, such as major depression were excluded from the study. The exclusion criteria were chosen because they influence cognitive abilities and thus also the study. The exclusion criteria were determined by a diagnostic interview.

### Measurements

The neuropsychological examination was performed in one test session, beginning with the mini mental state examination (MMSE) and followed by the neuropsychological test battery Vienna (NTBV) and took approximately 60 min. The NTBV includes different subtests, which can be classified into the domains attention, executive functioning, language and memory. Attention was assessed by the age concentration test (“Alters-Konzentrations-Test”, AKT) [20], the digit-symbol subtest of the German Wechsler adult intelligence scale-revised [21] and the symbol counting task from the cerebral insufficiency (CI) test [22]. The trail making test B (TMT B) [23] and the score difference of the trail making tests A (TMT A) and B were also used to determine attention. Executive functioning was evaluated by the subtests TMT A [23], the planning maze test from the *Nürnberger Alters Inventar Test Battery* [24] and the five-point test [25]. As well, the Stroop test from the *Nürnberger Alters Inventar Test Battery* [24], the interference test from the CI [22] and the phonematic verbal fluency (PWT) test [26] were applied to investigate executive functioning. The Boston naming test (BNT) [27] and semantic verbal fluency test (SWT) [26] were used to examine language. The domain memory was assessed using the verbal selective reminding test (VSRT) [28], with the subtests for immediate recall, total recall, delayed recall and recognition [29]. Only participants with scores higher than 23 on the MMSE at first examination were considered for the study. Verbal intelligence was assessed by the Wortschatz-Test (WST IQ) [30], which is often used with patients with dementia. The Beck depression inventory (BDI-II) [31] was used to measure symptoms of depression. It has 21 questions asking how often the person has been feeling in certain ways in the last 2 weeks. The rating is on a 4-point scale and scores above 13 are consistent with clinical depressive symptoms.

### Statistical analyses

The RCI methodology was performed with the Reliable Change Calculator, version 1.0 [12]. Mean test scores of the NTBV and their standard deviations for each diagnostic group and both examinations were used in this case to calculate the RCI, as suggested by Chelune et al. [32] using the formula

$$RCI=(\left(X_{2}-X_{1}\right)-(M_{2}-M_{1}))/SED$$

The individual test scores of a participant at both examinations are represented by X_2_ and X_1,_ while M_2_ and M_1_ characterize the mean score of the comparing group at the two examinations. To calculate the standard error of difference (SED), standard deviation (SD) from first examination and the rtt of the comparison HC group were used to compute the standard error of the mean (SEM).

$$SED=\sqrt{2}*SEM$$

$$SEM=SD^{2}*(1-rtt)$$

A change of ±1.645, which is significant, is shown.

In order to clarify the calculations, here is a case example in more detail. A patient from the AD group was compared to the HC group for the SWT subtest. First, the mean score and SD of the HC group were computed to calculate the confidence interval. The mean score of the SWT subtest at first examination was 75.15, with a SD of 13.94. At the follow-up examination, the mean score of the SWT subtest was 70.24, with a SD of 16.62 for the HC group. The rtt was assessed with a correlation of 0.69. The formula for SEM was applied by entering the SD and rtt from the HC group.

$$SEM=13.94*\left(1-0.69\right)=4.32$$

Next, the SED was calculated by including SEM in the formula.

$$SED=\sqrt{2}*4.32=2.94$$

A significant change of ±1.645 can be assumed [32].

To demonstrate a statistically significant change between both examinations, the RCI of the AD patient has to be higher or lower than the limit values. The patient chosen for the example reached a test score of 30 (X_1)_ at first examination and a test score of 26 (X_2_) at second examination in the SWT subtest. The RCI was calculated by including the mean score of the group and the individual patient’s score in the formula.

$$RCI=((26-30)-(70.24-75.15))/2.94=3.03$$

The RCI of 3.03 for this individual patient was compared with the limit values determined above. A higher value than the limiting value of 1.645 was found. Therefore, when this patient was compared to the HC group, deterioration in the SWT subtest could be seen. Mann-Whitney U-test analyses were conducted to measure differences in demographic and clinical characteristics between diagnostic groups.

## Results

The study included 358 patients aged 51–94 years old (*median* = 69 years, *SD* = 9.12 years), 175 were male (48.9%) and 183 were female (51.1%). They had between 6 and 24 years (*median* = 11.00, *SD* = 4.12) of education. Mann-Whitney U-test analyses showed no significant differences (*p* > 0.1) between the three groups in terms of verbal intelligence, depression symptoms and education. For more information about demographic and clinical baseline characteristics see Table 1. The mean interval between first and second examination was 25.96 months (*SD* = 11.28). Some participants converted to different diagnoses between first and second examination. A description of the rates of conversion across both examinations for all diagnostic groups is shown in Table 2. Neuropsychological test-retest data for healthy controls are presented in Table 3.

**Table 1:** Demographic and clinical characteristics

	HC (*N* = 46)	SCD (*N* = 42)	MCI (*N* = 270)	Total (*N* = 358)
Age (years)	61 (51–76)	66 (50–86)	70 (50–92)	69 (50–92)
Sex (m/w)	30/16	21/21	124/146	175/183
Education (years)	12 (6–24)	12 (8–22)	11 (8–22)	11 (6–24)
MMSE	29 (27–30)	29 (26–30)	28 (22–30)	28 (22–30)
BDI-II	2 (0–29)	8 (0–31)	9 (0–50)	8 (0–50)
WST-IQ	116 (90–139)	118 (88–133)	110 (77–140)	110 (77–140)

*Note.* median (range)

*HC* healthy controls,* SCD* subjective cognitive decline*, MCI* mild cognitive impairment, *MMSE* mini mental state examination, *BDI-II* Beck depression inventory, *WST-IQ* Wortschatztest Intelligenzquotient

**Table 2:** Rates of conversion across examinations in the subgroups

		Follow-up				Total
		HC	SCD	MCI	AD	
Baseline	HC	46 (100.0)	0	0	0	46
	SCD	0	25 (59.5)	17 (40.5)	0	42
	MCI	0	39 (14.4)	183 (67.8)	48 (17.8)	270
Total	–	46	64	200	48	358

*Note*. *N* (%)

*HC* healthy controls, *SCD* subjective cognitive decline*, MCI* mild cognitive impairment, *AD* Alzheimer’s disease

**Table 3:** Mean test scores and test-retest reliability (rtt) of healthy controls

Subtest	Baseline	Follow-up	rtt
AKT	28.33 ± 8.51	29.96 ± 11.01	0.59
AKT G/T	2.00 ± 0.54	2.07 ± 0.55	0.68
Digit-symbol	51.07 ± 11.88	48.65 ± 11.41	0.80
TMT B	83.59 ± 38.67	81.33 ± 45.17	0.72
C.I. symbols	18.41 ± 3.55	18.09 ± 4.10	0.41
TMT B—TMT A	50.02 ± 34.58	46.67 ± 40.26	0.67
*Language*			
SWT	75.15 ± 13.94	70.24 ± 16.62	0.76
BNT	14.72 ± 0.58	14.72 ± 0.58	0.61
*Memory*			
VSRT immediate	9.20 ± 1.87	8.48 ± 2.37	0.60
VSRT total	55.46 ± 9.08	55.30 ± 9.48	0.76
VSRT delayed	11.22 ± 2.75	11.89 ± 2.39	0.66
VSRT recognition	14.41 ± 0.93	13.92 ± 2.19	0.51
*Executive function*			
TMT A	33.59 ± 10.93	34.65 ± 10.93	0.61
PWT	41.85 ± 11.63	41.76 ± 10.74	0.69
5 Point	35.41 ± 8.62	34.70 ± 10.33	0.47
Stroop color	21.65 ± 4.80	21.85 ± 3.92	0.85
Stroop words	39.59 ± 9.55	39.59 ± 9.70	0.81
Stroop TT	0.84 ± 0.22	0.98 ± 0.24	0.67
Stroop difference	16.65 ± 6.51	15.59 ± 7.23	0.66
Planning maze	33.61 ± 14.60	27.98 ± 11.88	0.65
Planning maze TT	0.54 ± 0.22	0.64 ± 0.28	0.61
Interference C.I.	19.48 ± 5.01	20.70 ± 6.17	0.59
Interference C.I. TT	1.83 ± 0.41	1.72 ± 0.49	0.77

*AKT* “Alters-Konzentrations-Test”, *TT* total/time, *TMT B* trail making test version B, *C.I.* cerebral insufficiency, *TMT A* trail making test version A, *SWT* semantic fluency, *BNT* Boston naming test, *VSRT* verbal selective reminding test, *PWT* phonematic fluency

The RCI methodology was employed to assess the performance of each participant in each diagnostic group for all NTBV scores across both examinations, taking into consideration changes unrelated to cognitive impairments. The percentage of participants who showed a decline in NTBV subtests ranged from 0% to 18.8% for SCD group. For the MCI group, the deterioration in severe NTBV score ranged from 1.6% to 29.1%. Converters to AD showed a decline between 0% and 54.2%. The percentage of the SCD group revealing an improvement ranged from 0% to 73.4%; for MCI group, the range was from 0% to 25.1%. For the AD group, the improvement at second examination ranged from 0% to 44.0%. The results of mean scores and the SD of NTBV scores for the HC group at first and second examination, along with the individual comparisons between the HC group and SCD group, the MCI group and converters to AD, are presented in Table 4.

**Table 4:** Percentage of patients with significant decline, no deterioration and improvement in the SCD group, MCI group and AD group

		SCD			MCI			AD	
	**↓↓**	**↔**	**↑↑**	**↓↓**	**↔**	**↑↑**	**↓↓**	**↔**	**↑↑**
AKT	1.6	92.2	6.2	4.0	86.0	10.0	10.9	65.0	24.1
AKT G/T	7.8	90.6	1.6	5.0	93.5	1.5	11.1	88.9	0.0
Digit-symbol	4.8	93.6	1.6	5.2	91.2	3.6	11.5	77.0	11.5
TMT B	1.6	93.6	4.8	24.6	68.6	6.8	54.2	33.3	12.5
C.I. symbols	9.4	81.2	9.4	8.6	86.3	5.1	20.0	68.9	11.1
TMT B—TMT A	3.2	95.2	1.6	6.3	72.1	21.6	0.0	56.0	44.0
*Language*									
SWT	3.2	82.5	14.3	2.6	85.8	11.6	0.0	96.0	4.0
BNT	18.8	43.7	37.5	29.1	45.8	25.1	50.0	23.9	26.1
*Memory*									
VSRT immediate	6.3	81.2	12.5	10.1	84.4	5.5	26.1	73.9	0.0
VSRT total	10.9	82.9	6.2	16.6	78.9	4.5	17.4	76.1	6.5
VSRT delayed	10.9	86.0	3.1	19.1	78.4	2.5	26.1	71.7	2.2
VSRT recognition	0.0	100.0	0.0	19.6	74.4	6.0	52.2	34.8	13.0
*Executive function*									
TMT A	4.7	89.0	6.3	15.6	74.3	10.1	34.8	52.2	13.0
PWT	4.8	93.6	1.6	1.6	91.0	7.4	0.0	96.0	4.0
5 point	3.2	93.6	3.2	1.6	94.2	4.2	0.0	92.0	8.0
Stroop color	7.9	82.6	9.5	19.3	68.9	11.8	20.0	52.0	28.0
Stroop words	9.5	81.0	9.5	14.4	74.4	11.2	32.0	44.0	24.0
Stroop TT	12.9	87.1	0.0	11.7	88.3	0.0	20.0	80.0	0.0
Stroop difference	7.9	82.6	9.5	15.2	74.7	16.8	15.4	50.0	34.6
Planning maze	7.8	87.5	4.7	18.6	71.3	10.1	23.9	71.8	4.3
Planning maze TT	7.8	92.2	0.0	5.1	93.9	1.0	4.3	93.5	2.2
Interference C.I.	1.6	25.0	73.4	7.5	86.0	6.5	28.3	58.7	13.0
Interference C.I. TT	3.1	90.7	6.2	5.8	89.5	4.7	8.7	82.6	8.7

SCD group, MCI group, AD group

↓↓ Decline

↔ no deterioration

↑↑ improvement

*AKT* “Alters-Konzentrations-Test”, *TT* total/time, *TMT B* trail making test version B, *C.I.* cerebral insufficiency, *TMT A* trail making test version A, *SWT* semantic fluency, *BNT* Boston naming test, *VSRT* verbal selective reminding test, *PWT* phonematic fluency

## Discussion

The RCI methodology was used to predict the cognitive outcome of NTBV subtests for each patient. Looking at the results of the SCD group no changes could be observed. Deterioration was under 10.0% for most of the subtests of NTBV. Regarding the improvement of the SCD group, positive changes were not higher than 10.0% for most of the subtests. Exceptions were the BNT with 37.5% and the interference C.I. time with 73.4% improvement. In the MCI group, no differences between first and second examination were expected. Furthermore, the RCI for the MCI group, the percentage of patients exhibiting significant decline, was up to 29.1%. Improvements in this diagnostic group could also be seen. The reason for the deterioration in the SCD and MCI groups could be different states of disease progression in the diagnostic group. For example, some patients diagnosed with MCI showed cognitive impairments in one and others in multiple domains of the NTBV. In addition, lack of motivation, aging and misclassification can have an influence on the test results. The improvements can be attributed to practice effects, also a lack of motivation or attention in the first test could have yielded “improvement” in the retest. Other reasons may be the influence of mood or a possible treatment between the two test dates. For converters to AD, a deterioration higher than 10% in all subtests but 6 (SWT, PWT, 5 point test, planning maze test total/time, TMT B‑A difference, interference C.I. total/time) was observed. Deterioration for this diagnostic group ranged up to 54.2%. These results underline the cognitive decline of patients suffering from the progression of dementia. Nevertheless, there were also unexpected apparent improvements in the AD group for the subtests AKT, BNT, Stroop color, Stroop words, Stroop color word difference and TMT B‑A difference. In related samples, improvement in test performance can be reached by practice effects [33]; however, cognitive training or therapeutic intervention between both examinations can also have an influence on the improved test results.

A number of limitations should be considered when interpreting the study results. Patients with a MMSE score under 24 at second examination received the short version of the NTBV because they were not capable of filling in the long version of the test. In consequence, there are missing values, which leads to a reduction of the representativeness of the sample. Moreover, current mood, which may influence cognitive performance while testing and cognitive training between examinations to preserve cognitive functions, was not investigated in this study [14, 15, 34, 35]. This should be considered in future research.

The RCI methodology is able to compare the test scores of individuals to determine improvements or deteriorations. The cognitive stability and change of a patient play important roles in the diagnosis of cognitive dysfunction related to dementia. Knowing about the individual development of the disease can help to evaluate the treatment effect. This can delay cognitive deterioration and maintain patient independence.

## References

[1] Saxton J, Morrow L, Eschman A, Archer G, Luther J, Zuccolotto A (2009). Computer assessment of mild cognitive impairment. Postgrad Med.

[2] Lehrner J, Bodner T, Dal-Bianco P, Schmidt R, Lehrner J, Pusswald G, Fertl E, Strubreither W, Kryspin-Exner I (2011). Demenzsyndrome. Klinische Neuropsychologie.

[3] Jessen F, Wiese B, Bachmann C (2010). Prediction of dementia by subjective memory impairment effects of severity and temporal association with cognitive impairment. Arch Gen Psychiatry.

[4] Jessen F, Wolfsgruber S, Wiese B (2014). AD dementia risk in late MCI, in early MCI, and in subjective memory impairment. Alzheimers Dement.

[5] Drummond C, Coutinho G, Fonseca RP (2015). Deficits in narrative discourse elicited by visual stimuli are already present in patients with mild cognitive impairment. Front Aging Neurosci.

[6] Elias MF, Beiser A, Wolf PA, Au R, White RF, D’Agostino RB (2000). The preclinical phase of alzheimer disease: a 22-year prospective study of the Framingham cohort. Arch Neurol.

[7] Grober E, Lipton RB, Hall C, Crystal H (2000). Memory impairment on free and cued selective reminding predicts dementia. Neurology.

[8] Maseda A, Lodeiro-Fernández L, Lorenzo-López L, Núñez-Naveira L, Balo A, Millán-Calenti JC (2014). Verbal fluency, naming and verbal comprehension: three aspects of language as predictors of cognitive impairment. Aging Ment Health.

[9] Fabrigoule C, Rouch I, Taberly A (1998). Cognitive process in preclinical phase of dementia. Brain.

[10] Pusswald G, Moser D, Gleiß A (2013). Prevalence of mild cognitive impairment subtypes in patients attending a memory outpatient clinic-comparison of two modes of mild cognitive impairment classification. Results of the vienna conversion to dementia study. Alzheimers Dement.

[11] Small BJ, Herlitz A, Fratiglioni L, Almkvist O, Bäckman L (1997). Cognitive predictors of incident Alzheimer’s disease: a prospective longitudinal study. Neuropsychology.

[12] Hinton-Bayre AD (2010). Deriving reliable change statistics from test–retest normative data: comparison of models and mathematical expressions. Arch Clin Neuropsychol.

[13] Carrière I, Fourrier-Reglat A, Dartigues JF (2009). Drugs with anticholinergic properties, cognitive decline, and dementia in an elderly general population: the 3-city study. Arch Intern Med.

[14] Hill NT, Mowszowski L, Naismith SL, Chadwick VL, Valenzuela M, Lampit A (2016). Computerized cognitive training in older adults with mild cognitive impairment or dementia: a systematic review and meta-analysis. Am J Psychiatry.

[15] Ngandu T, Lehtisalo J, Solomon A (2015). A 2 year multidomain intervention of diet, exercise, cognitive training, and vascular risk monitoring versus control to prevent cognitive decline in at-risk elderly people (FINGER): a randomised controlled trial. Lancet.

[16] Jessen F, Amariglio RE, Van Boxtel M (2014). A conceptual framework for research on subjective cognitive decline in preclinical Alzheimer’s disease. Alzheimers Dement.

[17] Petersen RC (2004). Mild cognitive impairment as a diagnostic entity. J Intern Med.

[18] American Psychiatric Association (2003). Diagnostic and statistical manual of mental disorders (DSM-4).

[19] McKhann G, Drachman D, Folstein M, Katzman R, Price D, Stadlan EM (1984). Clinical diagnosis of Alzheimer’s disease: report of the NINCDS-ADRDA work group* under the auspices of Department of Health and Human Services Task Force on Alzheimer’s Disease. Neurology.

[20] Gatterer G (1990). Alters-Konzentrations-Test (AKT).

[21] Tewes U (1994). Hamburg-Wechsler-Intelligenztest für Erwachsene – Revision 1991 (HAWIE-R).

[22] Lehrl S, Fischer B (1997). Kurztest für Cerebale Insuffizienz (c.I.-Test).

[23] Reitan R (1979). Trail making test (TMT).

[24] Oswald WD, Fleischmann UM (1997). Das Nünberger-Alters-Inventar.

[25] Regard M, Strauss E, Knapp P (1982). Children’s production on verbal and non-verbal fluency tasks. Percept Mot Skills.

[26] Goodglass H, Kaplan P (1983). The assessment of aphasia and related disorders.

[27] Morris JC, Heyman A, Mohs RC (1989). The consortium to establish a registry for Alzheimer’s disease (CERAD): I. Clinical and neuropsychological assessment of Alzheimer’s disease. Neurology.

[28] Lehrner J, Gleiß A, Maly J, Auff E, Dal-Bianco P (2006). Der Verbale Selektive Reminding Test (VSRT). Ein Verfahren zur Überprüfung verbaler Gedächtnisfunktionen. Neuropsychiatrie.

[29] Youngjohn JR, Larrabee GJ, Crook TH (1991). First-last names and the grocery list selective reminding test: two computerized measures of everyday verbal learning. Arch Clin Neuropsychol.

[30] Schmidt K-H, Metzler P (1992). Wortschatztest: WST.

[31] Hautzinger M, Keller F, Kühner C (2006). Beck Depressions-Inventar (BDI-II).

[32] Chelune GJ, Naugle RI, Lueders H, Sedlak J, Awad IA (1993). Individual change after epilepsy surgery: practice effects and base-rate information. Neuropsychology.

[33] Foki T, Hitzl D, Pirker W (2017). Assessment of individual cognitive changes after deep brain stimulation surgery in Parkinson’s disease using the Neuropsychological Test Battery Vienna short version. Wien Klin Wochenschr.

[34] Boone KB (2009). Fixed belief in cognitive dysfunction despite normal neuropsychological scores: neurocognitive hypochondriasis?. Clin Neuropsychol.

[35] Marino SE, Meador KJ, Loring DW (2009). Subjective perception of cognition is related to mood and not performance. Epilepsy Behav.

